# Human Sclera Maintains Common Characteristics with Cartilage throughout Evolution

**DOI:** 10.1371/journal.pone.0003709

**Published:** 2008-11-12

**Authors:** Yuko Seko, Noriyuki Azuma, Yoriko Takahashi, Hatsune Makino, Toshiyuki Morito, Takeshi Muneta, Kenji Matsumoto, Hirohisa Saito, Ichiro Sekiya, Akihiro Umezawa

**Affiliations:** 1 Department of Reproductive Biology and Pathology, National Institute for Child and Health Development, Tokyo, Japan; 2 Department of Ophthalmology, National Center for Child Health and Development, Tokyo, Japan; 3 Section of Orthopaedic Surgery, Tokyo Medical and Dental University, Tokyo, Japan; 4 Department of Allergy and Immunology, National Institute for Child and Health Development, Tokyo, Japan; 5 Section of Cartilage Regeneration, Tokyo Medical and Dental University, Tokyo, Japan; University of Reading, United Kingdom

## Abstract

**Background:**

The sclera maintains and protects the eye ball, which receives visual inputs. Although the sclera does not contribute significantly to visual perception, scleral diseases such as refractory scleritis, scleral perforation and pathological myopia are considered incurable or difficult to cure. The aim of this study is to identify characteristics of the human sclera as one of the connective tissues derived from the neural crest and mesoderm.

**Methodology/Principal Findings:**

We have demonstrated microarray data of cultured human infant scleral cells. Hierarchical clustering was performed to group scleral cells and other mesenchymal cells into subcategories. Hierarchical clustering analysis showed similarity between scleral cells and auricular cartilage-derived cells. Cultured micromasses of scleral cells exposed to TGF-βs and BMP2 produced an abundant matrix. The expression of cartilage-associated genes, such as Indian hedge hog, type X collagen, and MMP13, was up-regulated within 3 weeks in vitro. These results suggest that human ‘sclera’-derived cells can be considered chondrocytes when cultured ex vivo.

**Conclusions/Significance:**

Our present study shows a chondrogenic potential of human sclera. Interestingly, the sclera of certain vertebrates, such as birds and fish, is composed of hyaline cartilage. Although the human sclera is not a cartilaginous tissue, the human sclera maintains chondrogenic potential throughout evolution. In addition, our findings directly explain an enigma that the sclera and the joint cartilage are common targets of inflammatory cells in rheumatic arthritis. The present global gene expression database will contribute to the clarification of the pathogenesis of developmental diseases such as high myopia.

## Introduction

The eye receives information from the outside as the retinal image, converting it into electrical signals for the brain, leading to visual perception. The retinal image is stabilized by the balance of intraocular pressure and the curvatures of the scleral and corneal envelope. In order to keep this balance, the rigidity of the sclera and the cornea are essential, especially the sclera must be rigid enough for the eyeball to be rotated by powerful extraocular muscles adhering to the sclera. The sclera and the corneal stroma that are anatomically continuous have common characteristics such as mechanical rigidity, and share a common origin, i.e., the neural crest. However, the cornea and the sclera are different in transparency: the cornea is completely transparent to produce a sharp image on the retina; the sclera is opaque to avoid the internal light scattering affecting the retinal image. This corneal transparency has been attributed to significant changes in the structure, especially of collagen fibrils, in the latter stages of development [1]. Multipotent progenitor/precursor cells of corneal stroma are identified from the mouse eye [2]. On the other hand, existence of multipotent progenitor/precursor cells in the sclera remains unclarified. Although the sclera does not contribute significantly to visual perception, scleral diseases such as refractory scleritis, scleral perforation and pathological myopia are considered incurable or difficult to cure.

Microarray analysis of murine scleral development [3] and global sequencing analysis from the human scleral cDNA library [4] have been reported. To clarify pathogenesis of developmental diseases such as high myopia, a database of genes expressed in the sclera of younger donors is important. We here demonstrate with a global expression database of human infant sclera that the sclera derived from the neural crest evolutionarily retains characteristics of cartilage.

## Results

### Isolation and cell culture of human scleral cells

**Figure 1:**
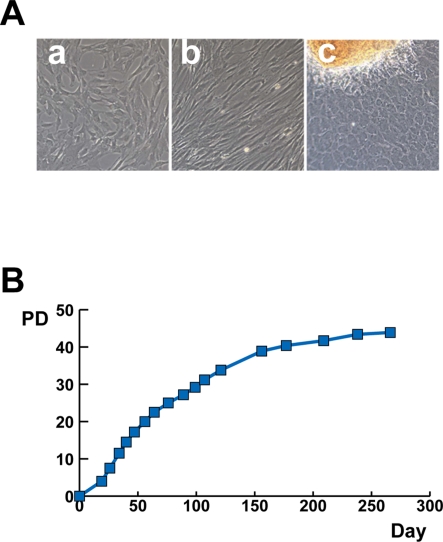
Proliferation of human ‘sclera’-derived cells. A. Photograph of primary cultured human ‘sclera’-derived cells by phase-contrast microscope. B. Growth curve of cultured human ‘sclera’-derived cells. Vertical axis indicates population doublings (PD) and horizontal axis indicates days after innoculation of human ‘sclera’-derived cells.

Scleral tissues were excised from surgical specimens collected during treatment for retinoblastoma. The scleral tissue was cut into smaller pieces and cultured in the growth medium. The scleral cells began growing out almost one week after the start of cultivation. Scleral cells exhibited a fibroblast-like spindle shape or polygonal shape in morphology when cultured in monolayer (Fig. 1A). The cells from PD 5 to PD 31 rapidly proliferated in culture, and propagated continuously (Fig. 1B). The cells stopped replicating and became broad and flat at PD 43 or 264 days, indicating that they had entered senescence. The morphological changes are PD-dependent.

### Global outlook by hierarchical clustering and PCA

**Figure 2:**
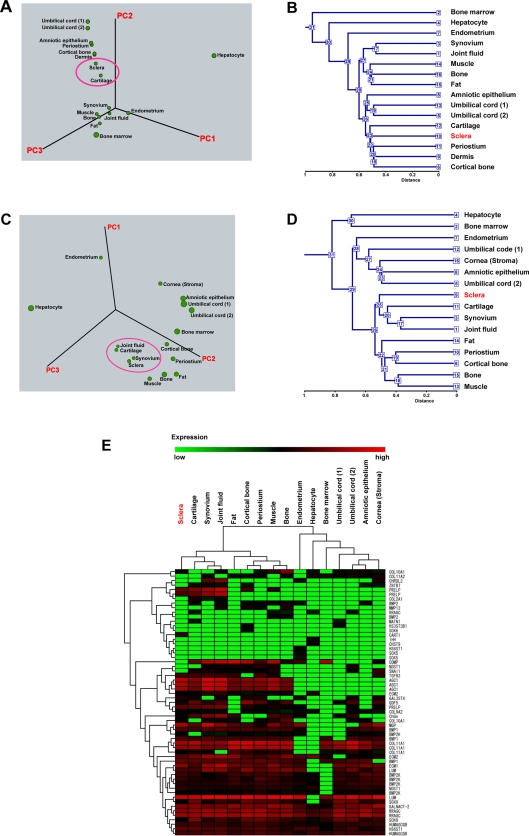
Global gene expression analysis of cultured human cells. A. Three-dimensional representation of PCA of gene expression levels (Human Genome U133 Plus 2.0: 54,675 probes). The gene expression data from scleral cells following one passage from the primary cultured cells (equivalent to appropriately 4 PDs) were used for PCA. Sclera and cartilage are positioned closely adjacent (shown in circle). B. Hierarchical clustering analysis based on the expression of all genes (Human Genome U133 Plus 2.0: 54,675 probes, NIA Array Analysis) shows similarity between scleral cells and chondrocytes. C. PCA of the cartilage-associated gene expression (Table S1). Sclera, cartilage, synovium, and joint fluid are positioned closely adjacent (shown in circle). D. Hierarchical clustering analysis based on expression levels of the cartilage-associated genes (NIA Array Analysis). Sclera, cartilage, synovium, and joint fluid are categorized into the same group. E. Hierarchical clustering analysis (TIGR MeV, see the Materials & Methods) with the heat map, based on expression levels of the cartilage-associated genes. Each row represents a gene; each column represents a cell population. Sclera, cartilage, synovium, and joint fluid are categorized into the same group. Cells derived from cartilage, synovium, and joint fluid are capable of generating cartilage in vivo [7], [34].

To clarify the specific gene expression profile of scleral cells, we compared the expression levels of 54,675 probes in the cultured scleral cells and other cultured cells (Table 1) using the Affymetrix GeneChip oligonucleotide arrays. We first performed hierarchical clustering and PCA on the expression pattern. PCA showed similarity between scleral cells and chondrocytes derived from elastic cartilage (Fig. 2A). Hierarchical clustering analysis based on all probes showed similarity between scleral cells and chondrocytes (Fig. 2B). This similarity led us to hypothesize that the scleral cells are chondrocytes when proliferated ex vivo, or have a chondrogenic potential. We then performed PCA from the expression data of cartilage-associated genes, including aggrecan, Sox9, and parathyroid hormone receptor (Table S1). These genes are categorized as “cartilage condensation” or “proteoglycan biosynthesis” according to Gene Ontology. PCA based on cartilage-associated genes demonstrated that scleral cells are grouped into the same category that includes chondrocytes, synovial cells, and synovial fluid-derived cells (Fig. 2C). The synovial cells and synovial fluid-derived cells used in this study have a strong chondrogenic potential [5]–[7]. Hierarchical clustering analysis based on the cartilage-associated genes also demonstrated that sclera, cartilage, synovium, and joint fluid are categorized into the same group (Fig. 2D, Fig. 2E, Fig. S1).

**Table 1:** Human cells analyzed in this study.

Title	Description
Bone marrow	Bone marrow-derived cell (P1)
Hepatocyte	Hepatocyte (P0)
Endometrium	Endometrial cell
Synovium	Synovium-derived cell (P1)
Joint fluid	Joint fluid-derived cell (P1)
Muscle	Muscle-derived cell (P1)
Bone	Cancellous bone-derived cell (P1)
Fat	Subcutaneus fat-derived cell (P1)
Amniotic epithelium	Amniotic epithelial cell (P4)
Umbilical cord (1)	Umbilical cord-derived cell (P0) (1)
Umbilical cord (2)	Umbilical cord-derived cell (P0) (2)
Cartilage	Auricular cartilage-derived cell (P1)
Sclera	Sclera-derived cell (P1)
Cornea (stroma)	Keratocyte (P1)
Periostium	Periostium-derived cell (P1)
Dermis	Dermal fibroblast (P2)
Cortical bone	Cortical bone-derived cell (P3)

Gene chip analysis was performed using RNAs from the cells obtained from each tissue. The cells obtained from bone marrow, liver, synovium, joint fluid, muscle, bone, and fat were cultivated as previously described [31]–[33]. Amniotic epithelial cells and umbilical cord-derived cells were cultured after each tissue was manually separated from the placenta and minced by surgical knife and scissors. Auricular cartilage-derived cells, periostium-derived cells, dermal fibroblasts, and cortical bone-derived cells started to be cultured after each tissue was manually separated from surgical specimens from patients with polydactyly or microtia. Keratocytes and scleral cells were obtained from corneal stroma and sclera (also see the Materials and Methods section). “Endometrium” was obtained from the homogenized endometrial cells under liquid nitrogen. All cells were harvested under signed informed consent, with the approval of the Ethics Committee of the National Institute for Child and Health Development, Tokyo. Signed informed consent was obtained from donors and the surgical specimens were irreversibly de-identified. All experiments handling human cells and tissues were performed in line with the Tenets of the Declaration of Helsinki. Global gene expression profiles of those cells are uploaded to GEO accession #GSE10934 at http://www.ncbi.nlm.nih.gov/geo/index.cgi.

P: passage. P0 and P1 represents primary cell culture and cell culture one passage after starting primary culture from tissues, respectively.

### Chondrogenesis of human scleral cells

**Figure 3:**
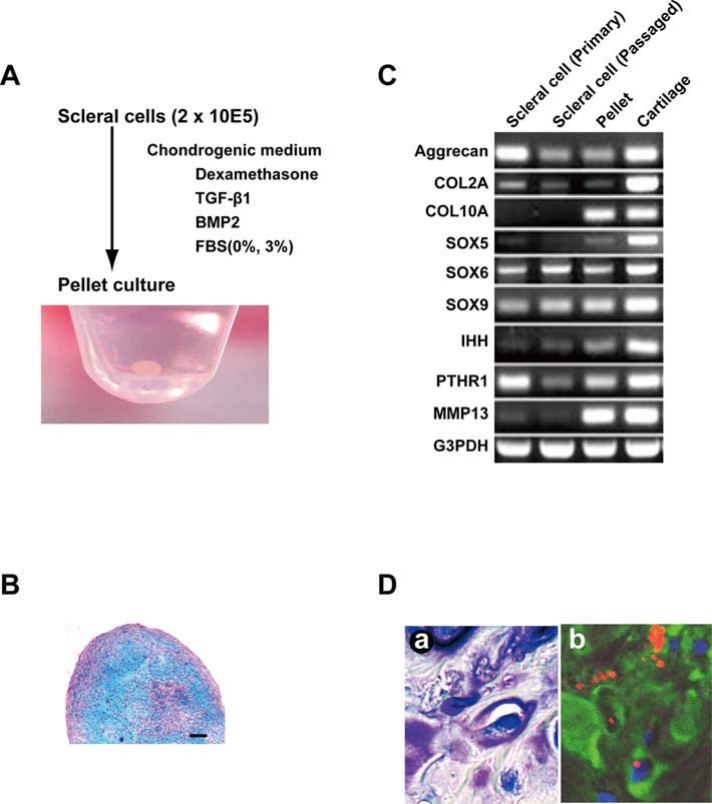
Chondrogenesis of human ‘sclera’-derived cells. A. In vitro chondrogenesis. ‘Sclera’-derived cells were centrifuged to make a pellet and cultured in chondrogenic medium for 4 weeks. Macroscopic feature is shown. B. Histological section of a pellet by micromass culture in a chondrogenic medium stained with alcian blue. Bar: 100 µm. C. Reverse transcriptase-PCR for cartilage-associated genes. Total RNAs were prepared from scleral cells at passage 0, at 10 population doublings, after in vitro chondrogenic induction, and normal cartilage as a positive control. D. Histological sections 4 weeks after transplantation of human scleral cells into cartilage defect of the knee in a rat. (a) Toluidin blue staining. (b) Immunohistochemistry. Human scleral cells were labeled with DiI (red). Nuclei were stained with DAPI (blue). Type II collagen was shown as green.

After reaching 70–80% sub-confluence, we started the micromass culture of scleral cells. Four weeks after culture in a chondrogenic medium containg TGF-β1 and BMP2, a pellet of human scleral cells exhibited a spherical shape (Fig. 3A). This pellet showed an alcian blue positive extracellular matrix, indicating that cultured micromasses of scleral cells exposed to TGF-β1 and BMP2 produce an abundant matrix (Fig. 3B). RT-PCR analysis demonstrated that scleral cells at passage 0 expressed aggrecan, COL2A, SOX5, SOX6, SOX9, and PTHR1 mRNAs (Fig. 3C). These expressions were maintained in the cells after 10 population doublings. After in vitro chondrogenesis of scleral cells, COL10A, SOX5, IHH, and MMP13 mRNA expressions increased. After human scleral cells labeled with DiI were implanted into a rat cartilage defect, the cells expressed type II collagen (Fig. 3D). These results demonstrated that human scleral cells retained chondrogenic potential both in vitro and in vivo.

## Discussion

### Tracing back of human scleral cells to chondrocytes through cultivation

**Figure 4:**
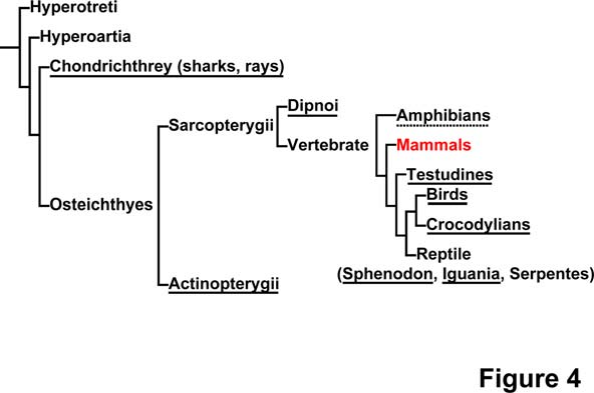
The distribution of scleral cartilage in vertebrates. The chondrogenic nature of the sclera is conserved across species. The figure is modified from Franz-Odendaal, TA, et al., 2006 [10]. Species that have cartilage in the sclera are underlined; species with either absence or presence of cartilage in the sclera, depending on family, are dot-underlined; species without cartilage in the sclera are non-underlined.

This study was undertaken to investigate if human sclera has a chondrogenic nature like chicken sclera [8], [9]. Bioinformatics of human scleral cells suggest similarity between scleral cells and chondrocytes, and this similarity may be attributed to evolution of the sclera (Fig. 4), that is, animals such as elasmobranch, teleost fish, amphibians, reptiles and birds incorporate the development of a cup of hyaline cartilage in the sclera [10]. Scleral cartilage is hypothesized to counter against the traction force of the extraocular muscle and against the accommodative force to move or deform the lens by intraocular muscles. In this paper, we employ the global gene expression approach to human scleral cells. As a result, scleral cells and chondrocytes are found to share common chondrogenic characteristics.

### Simulation of chondrogenic process during development

The phenotype of the differentiated chondrocyte is characterized by the synthesis, deposition, and maintenance of cartilage-specific extracellular matrix molecules, including type II collagen and aggrecan [11]–[13]. Three-dimensional culture is a prerequisite for exhibition of this chondrogenic phenotype in vitro since the phenotype of differentiated chondrocytes is unstable in culture and is rapidly lost during serial monolayer subculturing [14]–[16]. The expression pattern of cartilage-associated genes in sclera-derived cells after induction is consistent with that of chondrocytes during development (Fig. 3C, Fig. S2): a) Consistent expression of type II collagen and aggrecan, markers of early-phase chondrogenesis [17], [18] in sclera-derived cells, indicates that sclera-derived cells retain their chondrogenic nature as a default state; b) Induction of type X collagen and MMP13 genes after pellet formation of sclera-derived cells may simulate late-stage chondrogenesis. In addition, other chondrocyte-associated genes, such as sox5, IHH, and PTHR1 were also up-regulated. Sox5 functions as a transcription factor necessary for chondrogenesis [19], [20], IHH promotes chondrogenesis as a cytokine [21], and PTHR1 mediates parathyroid hormone signaling as a specific receptor [18]. These results suggest that ex vivo culture of sclera-derived cells simulates the developmental process of chondrogenesis. Despite the chondrogenic nature of sclera-derived cells, lack of cartilage in the sclera in humans may be attributed to cis- and trans-regulation of cartilage-associated gene(s), or an unclarified inhibitory mechanism that was altered during evolution (Fig. 4).

### Implication of chondrogenic nature of sclera in diseases

The fact that the gene expression pattern of the human fibrous sclera is similar to that of cartilage is interesting not only as comparative anatomy but also from a patho-etiological view point. The sclera and the joint cartilage are common targets for inflammatory cells in rheumatic arthritis [22], [23] or polychondritis [24], implying common proteins between the sclera and the synovium. Although the target protein(s) remains unclarified, our findings directly explain an enigma that both the sclera and the joint cartilage are affected in rheumatic arthritis. Furthermore, mutations in genes for type II and type XI collagen are a cause of Stickler syndrome [25], [26]. Patients with Stickler syndrome have joint deformity and severe high myopia due to an abnormality of the sclera. These affected lesions may be attributed to the chondrogenic nature of human sclera. In conclusion, our present study shows a chondrogenic potential of human sclera and explains the etiology of scleral disorders, at least in part. In addition, we would like to emphasize that the first database of gene expression in the human infant sclera (uploaded to GEO accession #GSE10934 at http://www.ncbi.nlm.nih.gov/geo/index.cgi) may contribute to the elucidation of scleral diseases in the future.

## Materials and Methods

### Isolation and cell culture of human scleral cells

Scleral tissues were excised from surgical specimens as a therapy of retinoblastoma, under signed informed consent, with the approval (approval number, #156) of the Ethics Committee of the National Institute for Child and Health Development, Tokyo. Signed informed consent was obtained from donors, and the surgical specimens were irreversibly de-identified. All experiments handling human cells and tissues were performed in line with the Tenets of the Declaration of Helsinki. The scleral pieces were cut into smaller pieces and cultured in the growth medium (GM): Dulbecco's modified Eagle's medium (DMEM)/Nutrient mixture F12 (1:1) with high glucose supplemented with 10% fetal bovine serum, insulin-transferrin-selenium, and MEM-NEAA (GIBCO).

### Oligonucleotide microarray

Total RNAs were isolated from cultured scleral cells in the growth medium without any induction of differentiation to perform the gene chip analysis. Total RNA was extracted from a total of 5×10^6^ cultured human scleral cells and other mesenchymal cells (Table 1) using RNeasy Plus mini-kit® (Qiagen, Maryland, USA) according to the manufacturer's instructions. A comprehensive expression analysis was performed using 2 µg of total RNA from each sample and GeneChip® Human Genome U133 plus 2.0 probe arrays (Affymetrix, Santa Clara, CA) according to the manufacturer's instructions. To normalize the variations in staining intensity among chips, the ‘Signal’ values for all probes on a given chip were divided by the median value for expression of all genes on the chip. To consider genes containing only a background signal, probes were eliminated only if the ‘Signal’ value was less than 10, or the Detection call was ‘Absent’ in any sample using GeneSpring software version 7.2 (Agilent Technologies, Palo Alto). The gene chip analysis was carried out on 8 independent scleral cultures.

### Hierarchical clustering and principal component analysis (PCA)

To analyze the gene expression data in an unsupervised manner by gene chip array, we used hierarchical clustering and principal component analysis (NIA Array; http://lgsun.grc.nia.nih.gov/ANOVA/ [27], TIGR MeV; http://www.tm4.org/mer.html [28]). The hierarchical clustering techniques classify data by similarity and the results are represented by dendrogram. PCA is a multivariate analysis technique which finds major patterns in data variability. Hierarchical clustering and PCA were performed on the data of gene chip analysis (a single assay for each sample) to group scleral cells and other mesenchymal cells into subcategories (Table 1).

### In vitro chondrogenesis

Two hundred thousand scleral cells were placed in a 15-ml polypropylene tube (Becton Dickinson) and centrifuged for 10 minutes. The pellet was cultured in DF-C medium™ containing 0.1 µM dexamethasone, 1 mM sodium pyruvate, 0.17 mM ascorbic acid-2-phosphate, 0.35 mM proline, 6.25 µg/ml bovine insulin, 6.25 µg/ml transferrin, 6.25 µg/ml selenous acid, 5.33 µg/ml linoleic acid, 1.25 mg/ml BSA, 5 ng/ml TGF-β1, 5 ng/ml BMP2, and 3% fetal bovine serum (TOYOBO). The medium was replaced every 3 to 4 days for 28 days. For microscopy, the pellets were embedded in paraffin, cut into 5-µm sections, and stained with alcian blue [29], [30].

### In vivo chondrogenesis

Under anesthesia, full thickness cartilage defects were created in the trochlear groove of the femur in SD rats. The defects were filled with DiI-labeled human scleral cells. The rats were returned to their cages after the operation and allowed to move freely. Animals were sacrificed with an overdose of sodium pentobarbital at 4 weeks after the operation. Specimens were dissected and embedded in paraffin. The sections were stained with toluidine blue and immunohistochemically stained with anti-type II collagen antibodies (clone F-57, DAIICHI FINE CHEMICAL, Co. Ltd., Toyama, Japan). All animals received humane care in compliance with the “Principles of Laboratory Animal Care” formulated by the National Society for Medical Research and the “Guide for the Care and Use of Laboratory Animals” prepared by the Institute of Laboratory Animal Resources and published by the US National Institutes of Health (NIH Publication No. 86-23, revised 1985). The operation protocols were accepted by the Laboratory Animal Care and Use Committee of the Research Institute for Child and Health Development (2003-002).

### Reverse transcriptase-PCR

Total RNA was isolated with an RNeasy Plus mini-kit. Cartilage pellets were digested with 3 mg/ml Collagenase D for 3 hours at 37°C.

The following PCR primer sets were used for cartilage-associated genes: aggrecan, sense (5′-TACACTGGCGAGCACTGTAAC -3′) and antisense (5′-CAGTGGCCCTGGTACTTGTT-3′), product size, 71 bp; collagen, type II, alpha 1, sense (5′-TTCAGCTATGGAGATGACAATC -3′) and antisense (5′-AGAGTCCTAGAGTGACTGAG -3′), product size, 472 bp; collagen, typeX, alpha 1, sense (5′-CACCTTCTGCACTGCTCATC-3′) and antisense (5′-GGCAGCATATTCTCAGATGGA-3′), product sizem, 104 bp; SOX5, sense (5′-AGCCAGAGTTAGCACAATAGG -3′) and antisense (5′-CATGATTGCCTTGTATTC -3′), product size, 619 bp; SOX6, sense (5′-ACTGTGGCTGAAGCACGAGTC -3′) and antisense (5′-TCCGCCATCTGTCTTCATACC -3′), product size, 562 bp; SOX9, sense (5′-GTACCCGCACTTGCACAAC-3′) and antisense (5′-TCGCTCTCGTTCAGAAGTCTC-3′), product size 72 bp; Indian hedgehog homolog (IHH), sense (5′-TGCATTGCTCCGTCAAGTC-3′) and antisense (5′-CCACTCTCCAGGCGTACCT-3′), product size 88 bp; parathyroid hormone receptor 1(PTHR1), sense (5′-CCTGAGTCTGAGGAGGACAAG-3′) and antisense (5′-CACAGGATGTGGTCCCATT-3′), product size 86 bp; matrix metallopeptidase 13 (MMP13), sense (5′-CCAGTCTCCGAGGAGAAACA-3′) and antisense (5′-AAAAACAGCTCCGCATCAAC-3′),product size, 85 bp, and GAPDH, sense (5′- GCTCAGACACCATGGGGAAGGT-3′) and antisense (5′-GTGGTGCAGGAGGCATTGCTGA-3′), product size, 474 bp.

## Supporting Information

Figure S1 Global gene expression analysis of cultured human cells. Hierarchical clustering analysis based on expression levels of the cartilage-associated genes (NIA Array Analysis). We performed gene chip analysis (a single assay for each analysis) for eight independent primary scleral cultures from five patients (donors). We started eight independent cultures from three different scleral sites of Donor 2 (e.g. the anterior site 1.5 mm apart from the limbs, the middle part, and the posterior part), 2 different scleral sites of Donor 5, and three scleral sites of Donor 1, 3, and 4. We performed hierarchical clustering analysis, using these independent cultures and obtained consistent results, that is, “sclera”-derived cells are categorized into one sub-group. Furthermore, the sclera, cartilage, synovium, and joint fluid are categorized into the same group.
(0.07 MB PDF)

Figure S2 Cartilage-associated gene expressions in cultured fibroblasts derived from the dermis and the sclera. Cartilage-associated gene expressions by RT-PCR in cultured fibroblasts derived from the dermis and the sclera. Aggrecan, COL2A, IHH and PTHR mRNA expressions were clearly stronger in the scleral fibroblasts compared to the dermal fibroblasts, indicating that chondrogenic nature could be specific for the sclera among collagenous tissues.
(0.01 MB PDF)

Table S1 Cartilage-associated genes
(0.01 MB PDF)
