# Supplementary figures and images for: Human Sclera Maintains Common Characteristics with Cartilage throughout Evolution
Source: PLoS One. 2008 Nov 12;3(11):e3709. doi: 10.1371/journal.pone.0003709 (PMC2579486; doi:10.1371/journal.pone.0003709)

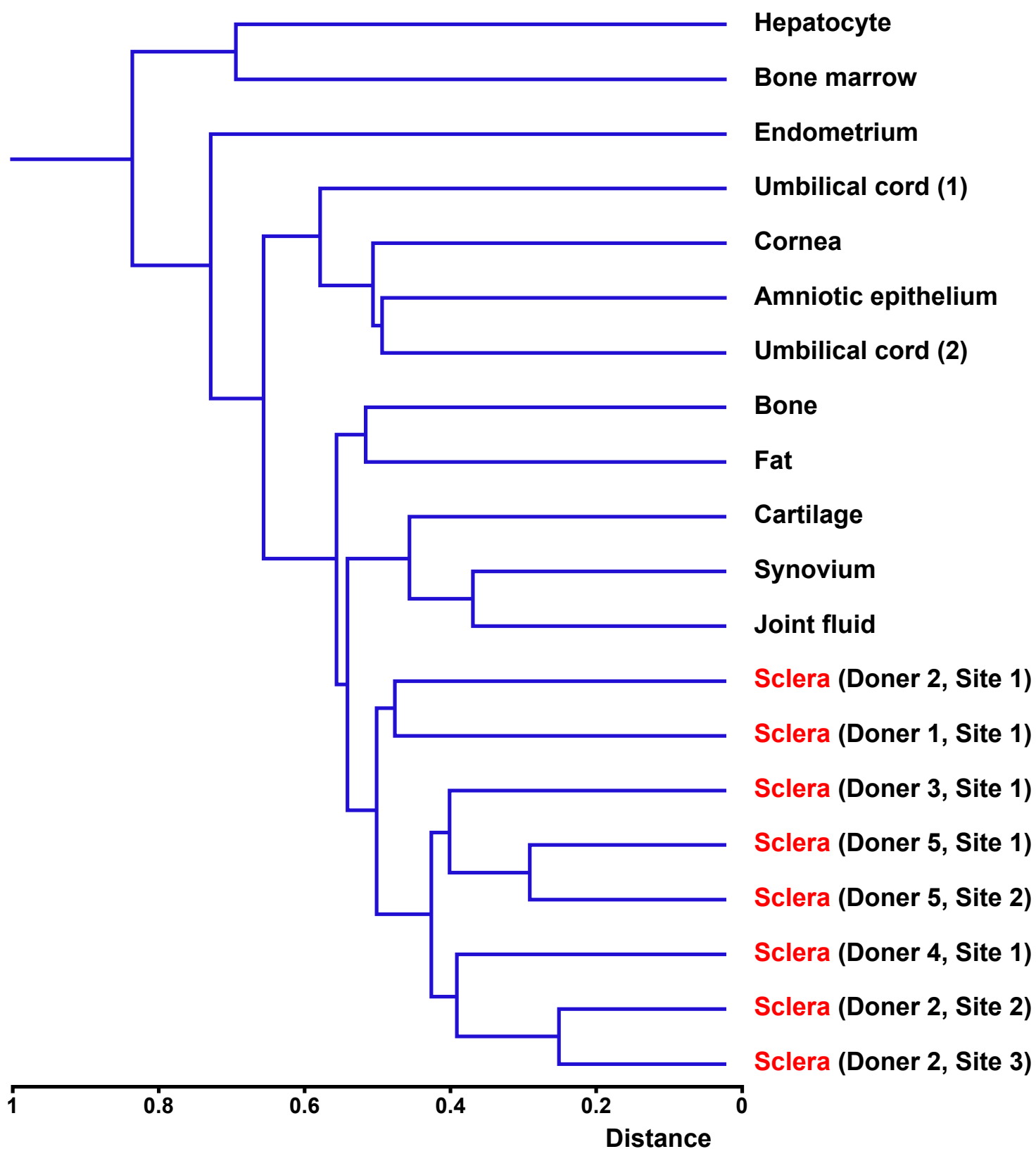

**Figure S1**

Supplement: Figure S1 — Global gene expression analysis of cultured human cells. Hierarchical clustering analysis based on expression levels of the cartilage-associated genes (NIA Array Analysis). We performed gene chip analysis (a single assay for each analysis) for eight independent primary scleral cultures from five patients (donors). We started eight independent cultures from three different scleral sites of Donor 2 (e.g. the anterior site 1.5 mm apart from the limbs, the middle part, and the posterior part), 2 different scleral sites of Donor 5, and three scleral sites of Donor 1, 3, and 4. We performed hierarchical clustering analysis, using these independent cultures and obtained consistent results, that is, “sclera”-derived cells are categorized into one sub-group. Furthermore, the sclera, cartilage, synovium, and joint fluid are categorized into the same group. (0.07 MB PDF) [file pone.0003709.s001.pdf]

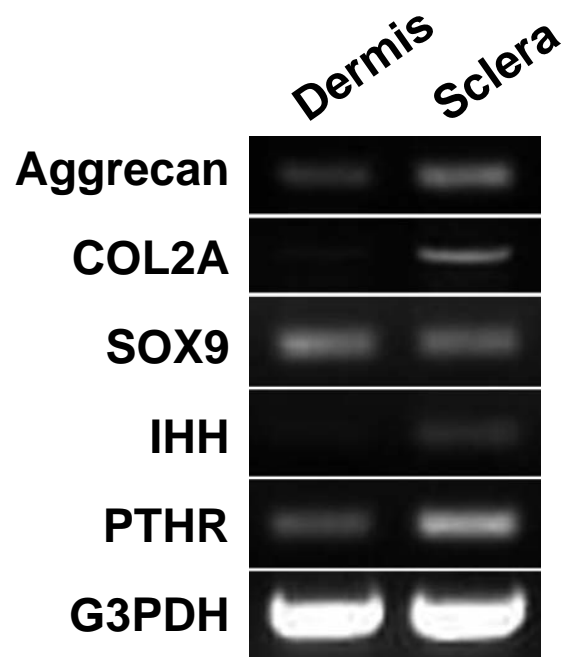

**Figure S2**

Supplement: Figure S2 — Cartilage-associated gene expressions in cultured fibroblasts derived from the dermis and the sclera. Cartilage-associated gene expressions by RT-PCR in cultured fibroblasts derived from the dermis and the sclera. Aggrecan, COL2A, IHH and PTHR mRNA expressions were clearly stronger in the scleral fibroblasts compared to the dermal fibroblasts, indicating that chondrogenic nature could be specific for the sclera among collagenous tissues. (0.01 MB PDF) [file pone.0003709.s002.pdf]
