# Supplementary material for: Human Sclera Maintains Common Characteristics with Cartilage throughout Evolution
Source: PLoS One. 2008 Nov 12;3(11):e3709. doi: 10.1371/journal.pone.0003709 (PMC2579486; doi:10.1371/journal.pone.0003709)
Supplement: Table S1 — Cartilage-associated genes (0.01 MB PDF) [file pone.0003709.s003.pdf]

Table S1. Cartilage-associated genes

Systematic	Common	Description	Genbank
1554950_at	AGC1	aggrecan 1	BC036445
205679_x_at	AGC1	aggrecan 1	NM_013227
207692_s_at	AGC1	aggrecan 1	NM_001135
217161_x_at	AGC1	aggrecan 1	X17406
202701_at	BMP1	bone morphogenetic protein 1	NM_006129
205574_x_at	BMP1	bone morphogenetic protein 1	NM_001199
206725_x_at	BMP1	bone morphogenetic protein 1	NM_006128
207595_s_at	BMP1	bone morphogenetic protein 1	NM_006132
205289_at	BMP2	bone morphogenetic protein 2	AA583044
205290_s_at	BMP2	bone morphogenetic protein 2	NM_001200
214716_at	BMP2K	BMP2 inducible kinase	AW504018
219546_at	BMP2K	BMP2 inducible kinase	NM_017593
226853_at	BMP2K	AU145366 HEMBA1 Homo sapiens cDNA clone HEMBA1004631 3', mRNA sequence.	AU145366
232688_at	BMP2K	AU144829 HEMBA1 Homo sapiens cDNA clone HEMBA1003086 3', mRNA sequence.	AU144829
37170_at	BMP2K	BMP2 inducible kinase	AB015331
59644_at	BMP2K	at10e09.x1 Barstead aorta HPLRB6 Homo sapiens cDNA clone IMAGE:2354728 3', mRNA sequence.	AI735391
206837_at	CART1	cartilage paired-class homeoprotein 1	NM_006982
219049_at	ChGn	chondroitin beta1,4 N-acetylgalactosaminyltransferase	NM_018371
223987_at	CHRDL2	chordin-like 2	AF332891
221065_s_at	CHST8	carbohydrate (N-acetylgalactosamine 4-0) sulfotransferase 8	NM_022467
223737_x_at	CHST9	carbohydrate (N-acetylgalactosamine 4-0) sulfotransferase 9	AF239821
224400_s_at	CHST9	carbohydrate (N-acetylgalactosamine 4-0) sulfotransferase 9	AF332473
205941_s_at	COL10A1	tc30d11.x1 Soares_total_fetus_Nb2HF8_9w Homo sapiens cDNA clone IMAGE:2066133 3'	AI376003

217428_s_at	COL10A1	H.sapiens type X collagen gene.	X98568
204320_at	COL11A1	collagen, type XI, alpha 1	NM_001854
229271_x_at	COL11A1	collagen, type XI, alpha 1	BG028597
37892_at	COL11A1	collagen, type XI, alpha 1	J04177
213870_at	COL11A2		AL031228
216993_s_at	COL11A2		U32169
213492_at	COL2A1	collagen, type II, alpha 1	X06268
217404_s_at	COL2A1	collagen, type II, alpha 1	X16468
1555527_at	COL9A1	collagen, type IX, alpha 1	BC015409
222008_at	COL9A1	collagen, type IX, alpha 1	NM_001851
243932_at	COL9A1	collagen, type IX, alpha 1	AI286254
213622_at	COL9A2	collagen, type IX, alpha 2	AI733465
231264_at	COL9A2		AL050341
232542_at	COL9A2	collagen, type IX, alpha 2	AU145185
204724_s_at	COL9A3	collagen, type IX, alpha 3	NM_001853
237427_at	COL9A3	collagen, type IX, alpha 3	AI678209
205713_s_at	COMP	cartilage oligomeric matrix protein	NM_000095
209365_s_at	ECM1	extracellular matrix protein 1	U65932
1568779_a_at	ECM2	extracellular matrix protein 2, female organ and adipocyte specific	AI473096
206101_at	ECM2	extracellular matrix protein 2, female organ and adipocyte specific	NM_001393
1553257_at	GAL3ST3	beta-galactose-3-O-sulfotransferase 3	AY026481
219815_at	GAL3ST4	beta-galactose-3-O-sulfotransferase, 4	NM_024637
218871_x_at	GALNACT-2	chondroitin sulfate GalNAcT-2	NM_018590
206614_at	GDF5	growth differentiation factor 5 (cartilage-derived morphogenetic protein-1)	NM_000557
221062_at	HS3ST3B1	heparan sulfate (glucosamine) 3-O-sulfotransferase 3B1	NM_006041

206997_s_at	HS6ST1	heparan sulfate 6-O-sulfotransferase 1	NM_004807
225263_at	HS6ST1	heparan sulfate 6-O-sulfotransferase 1	BC001196
202689_at	HUMAGC GB	chromosome 3p21.1 gene sequence	NM_013286
226987_at	HUMAGC GB	chromosome 3p21.1 gene sequence	W68720
215420_at	IHH	Indian hedgehog homolog (Drosophila)	BE869172
229358_at	IHH	Indian hedgehog homolog (Drosophila)	AA628967
201744_s_at	LUM	lumican	NM_002345
229554_at	LUM	lumican	AI141861
206904_at	MATN1	matrilin 1, cartilage matrix protein	M55683
206905_s_at	MATN1	matrilin 1, cartilage matrix protein	NM_002379
202291_s_at	MGP	matrix Gla protein	NM_000900
205959_at	MMP13	matrix metalloproteinase 13 (collagenase 3)	NM_002427
1554010_at	NDST1	N-deacetylase/N-sulfotransferase (heparan glucosaminyl) 1	BC012888
202608_s_at	NDST1	N-deacetylase/N-sulfotransferase (heparan glucosaminyl) 1	NM_001543
204223_at	PRELP	proline arginine-rich end leucine-rich repeat protein	NM_002725
228224_at	PRELP	proline arginine-rich end leucine-rich repeat protein	AA573140
37022_at	PRELP	proline-arginine-rich end leucine-rich repeat protein PRELP; Human prolargin (PRELP) gene, exon 3 and complete cds.	U41344
218088_s_at	RRAGC	Ras-related GTP binding C	NM_022157
222514_at	RRAGC	Ras-related GTP binding C	AK023373
242531_at	RRAGC	Ras-related GTP binding C	H56010
219480_at	SNAI1	snail homolog 1 (Drosophila)	NM_005985
1569638_at	SOX5	SRY (sex determining region Y)-box 5	BC014929
207336_at	SOX5	SRY (sex determining region Y)-box 5	NM_006940
216561_x_at	SOX5	Homo sapiens SOX-29 protein (SOX29) gene, partial cds.	AF032454
238285_at	SOX5	SRY (sex determining region Y)-box 5	BE467463

1563454_at	SOX6	SRY (sex determining region Y)-box 6	AL833302
1570486_at	SOX6	SRY (sex determining region Y)-box 6	BC029429
223865_at	SOX6	SRY (sex determining region Y)-box 6	AF309034
224178_s_at	SOX6	SRY (sex determining region Y)-box 6	AL136780
202935_s_at	SOX9	SRY (sex determining region Y)-box 9 (campomelic dysplasia, autosomal sex-reversal)	AI382146
202936_s_at	SOX9	SRY (sex determining region Y)-box 9 (campomelic dysplasia, autosomal sex-reversal)	NM_000346
1555540_at	TGFB3	transforming growth factor, beta 3	BC018503
209747_at	TGFB3	transforming growth factor, beta 3	J03241
213303_x_at	ZBTB7	zinc finger and BTB domain containing 7	AF097916
